# Wogonoside inhibits IL-1β induced catabolism and hypertrophy in mouse chondrocyte and ameliorates murine osteoarthritis

**DOI:** 10.18632/oncotarget.18374

**Published:** 2017-06-06

**Authors:** Qian Tang, Gang Zheng, Zhenhua Feng, Minji Tong, Jianxiang Xu, Zhiyan Hu, Ping Shang, Yu Chen, Chenggui Wang, Yiting Lou, Deheng Chen, Di Zhang, Majid Nisar, Xiaolei Zhang, Huazi Xu, Haixiao Liu

**Affiliations:** ^1^ Department of Orthopaedic Surgery, The Second Affiliated Hospital and Yuying Children’s Hospital of Wenzhou Medical University, 325027 Wenzhou, China; ^2^ Zhejiang Provincial Key Laboratory of Orthopaedics, 325027 Wenzhou, China; ^3^ Department of Rehabilitation, The Second Affiliated Hospital and Yuying Children’s Hospital of Wenzhou Medical University, 325027 Wenzhou, China; ^4^ Department of Anesthesiology, The Second Affiliated Hospital and Yuying Children's Hospital of Wenzhou Medical University, 325027 Wenzhou, China

**Keywords:** wogonoside, osteoarthritis, NF-κB, HIF-2α, hypertrophy

## Abstract

The inflammatory environment is correlated with extracellular matrix (ECM) degradation and chondrocyte hypertrophy in the development of osteoarthritis (OA). Previous studies have reported the anti-inflammatory effects of wogonoside in several diseases. In the present study, we investigated the protective effects of wogonoside in relation to the development of OA and delineated the potential mechanism. *In vitro*, wogonoside decreased the production of pro-inflammatory cytokines like Nitric oxide (NO), prostaglandin E2 (PGE_2_), tumor necrosis factor alpha (TNF-α), and interleukin-6 (IL-6). It also inhibited the expression of cyclooxygenase-2 (COX-2) and inducible nitric oxide synthase (iNOS) both at gene and protein levels. Wogonoside also inhibited hypertrophy and the generation of vascular endothelial growth factor (VEGF) in interleukin-1β (IL-1β)-induced chondrocytes. Moreover, wogonoside promoted the expression of anabolic factors Sox-9, type two collagen and aggrecan while inhibiting the expression of catabolic factors such as matrix metalloproteinases (MMPs) and thrombospondin motifs 5 (ADAMTS-5) in mouse chondrocytes. Mechanistically, we found that wogonoside inhibited nuclear factor kappa B/ hypoxia-inducible factor two alpha (NF-κB/HIF-2α) activation via the phosphatidylinositol 3 kinase (PI3K) /AKT pathway. The protective effects of wogonoside were also observed *in vivo* and the pharmacokinetic results of wogonoside indicated that good systemic exposure was achievable after oral administration of wogonoside. In conclusion, our stduy demonstrates that wogonoside attenuates IL-1β-induced ECM degradation and hypertrophy in mouse chondrocytes via suppressing the activation of NF-κB/HIF-2α by the PI3K/AKT pathway. Moreover, wogonoside ameliorates OA progression *in vivo*, indicating that wogonoside may serve as a promising therapeutic agent for the treatment of OA.

## INTRODUCTION

Osteoarthritis (OA), a painful degenerative joint disease characterized by articular cartilage loss, subchondral bone remodeling and inflammation of the synovium, causes progressive disabilities in the elderly due to its irreversible outcomes [1, 2]. Until now, the pathogenesis of OA is unclear and no effective therapeutic agent exists for OA [1]. However, it is generally accepted that the chondrocyte, the only cell type in articular cartilage, is a target of many potential risk factors of OA, including mechanical stress, senescence and inflammatory factors [3–5].

The extracellular matrix (ECM) mainly consists of type II collagen and aggrecan, the major components of normal cartilage that allow articular cartilage to adjust to biomechanical forces during joint movement [6]. The ECM is secreted and maintained by chondrocytes and is regulated by the Sox-9 gene, a key transcription factor for ECM homeostasis [7, 8]. There is now a general consensus that inflammation plays an important role in the OA process, contributing to the shift of chondrocyte phenotype and degradation of the ECM [8–10]. Inflammatory cytokines such as interleukin-1 beta (IL-1β), interleukin-6 and tumor necrosis factor-alpha (TNF-α) produced by activated synoviocytes, mononuclear cells or by articular cartilage itself are strongly related to the pathophysiology of osteoarthritis [11]. Among these cytokines, the IL-1β exert its inflammatory effects by significantly increasing the secretion of pro-inflammatory factors and catabolic factors such as prostaglandin E2 (PGE_2_), nitric oxide (NO), thrombospondin motifs (ADAMTS), and matrix metalloproteinases (MMPs) to destroy the ECM [12–14]. In contrast, the IL-1β stimulus can also lead to the hypertrophic-like conversion of chondrocytes [8]. As a result, OA chondrocytes are unable to sustain cartilage homeostasis and fail to replace components of the ECM, particularly collagen.

The transcription factor nuclear factor kappa B (NF-κB) is a key regulator of the production of pro-inflammatory factors and catabolic factors [15]. Activation of the NF-κB pathway includes the phosphorylation of p65 and IκBα in the cytoplasm and ultimately transfer p65 from the cytoplasm entering the nucleus [16]. The PI3K/AKT pathway is one of most widely studied upstream signaling pathways of NF-κB and is a significant target of chondrocyte inflammation and apoptosis [17]. Moreover, hypoxia-inducible factor two alpha (HIF-2α), one of major angiogenic and hypertrophic factors in OA chondrocytes, is induced by well-known mediators such as IL-1β and TNF-α, which directly target various catabolic factors including collagen X, ADAMTS-5, MMPs, and vascular endothelial growth factor (VEGF) to trigger cartilage destruction [18–21]. Additionally, it is worth noting that the HIF-2α-mediated destructive effects of chondrocytes can be initiated by NF-κB activation [19–21], suggesting a possible therapeutic target to unite anti-inflammation, anti-hypertrophy and anti-catabolism of the ECM to treat degenerative cartilage.

Currently, there are no effective target drugs for the treatment of OA [22]. Therefore, a safer and more effective drug is urgently needed to treat OA. Wogonoside, an individual component extracted from Scutellaria baicalensis Georgi, exhibits anti-oxidant, anti-tumor, anti-thrombotic, and anti-inflammatory effects [23–26]. Wogonoside not only affects the translation of inducible nitric oxide synthase (iNOS) and cyclooxygenase 2 (COX-2) but also inhibits lipopolysaccharide (LPS)-induced TNF-α and IL-6 expression in RAW264.7 cells [26]. In addition, wogonoside exerts a protective effect in inflammation-induced angiogenesis in endothelial cells [27]. The anti-inflammatory effects of wogonoside in tumors have been associated with inhibition of the PI3K/AKT/NF-κB pathway [25]. After NF-κB activating, the transcriptional level of HIF-2α is up-regulated, which is involved in the hypertrophic shift and VEGF generation in OA chondrocytes [18]. We investigated, therefore, the potential of wogonoside as a protective agent for OA treatment by inhibiting inflammation, ECM degradation, hypertrophy, and angiogenesis in chondrocytes via the cross-talk between the PI3K/AKT/NF-κB and HIF-2α pathways and by delaying the development of OA in a rodent model of destabilization of the medial meniscus (DMM).

## RESULTS

### Phenotypic determination of primary chondrocytes

**Figure 1:**
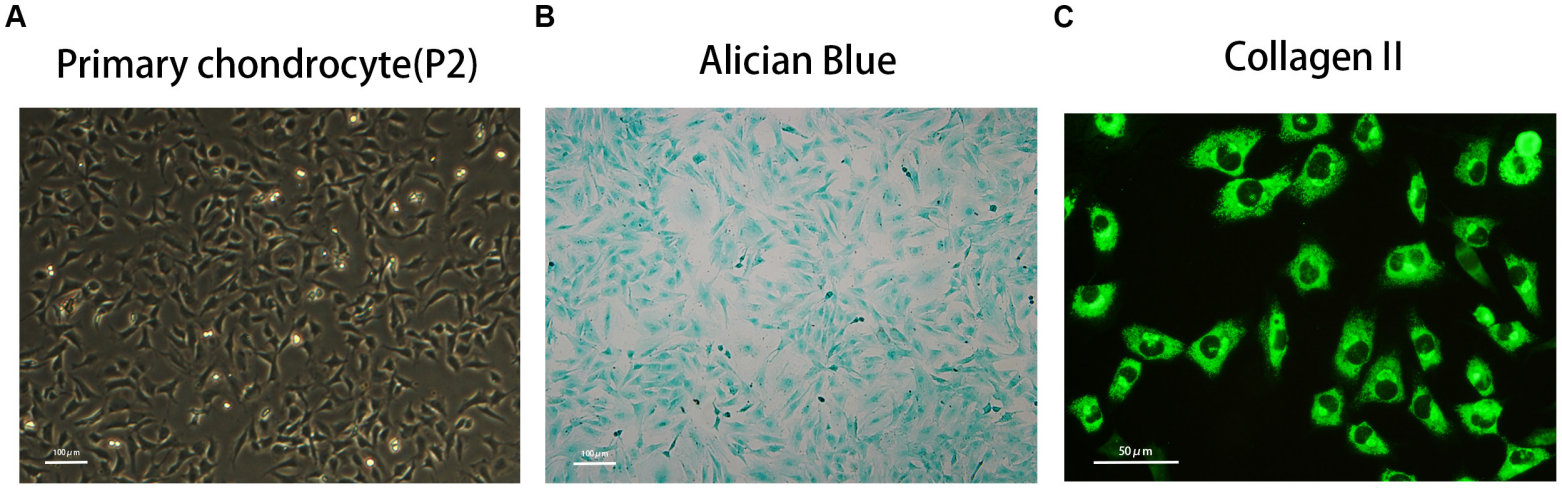
Phenotype determination of mice primary chondrocyte **(A)** The morphology of normal passage two primary chondrocytes. **(B)** Alcian blue stain was performed to stain the proteoglycan in chondrocytes. **(C)** The representative type two collagen was detected by immunofluorescence.

The morphology of primary chondrocytes (passage 2) is shown in Figure 1A. The phenotype was determined by Alcian blue staining and immunofluorescence of type II collagen staining, as shown in Figure 1B and 1C.

### Effects of wogonoside on chondrocyte viability

**Figure 2:**
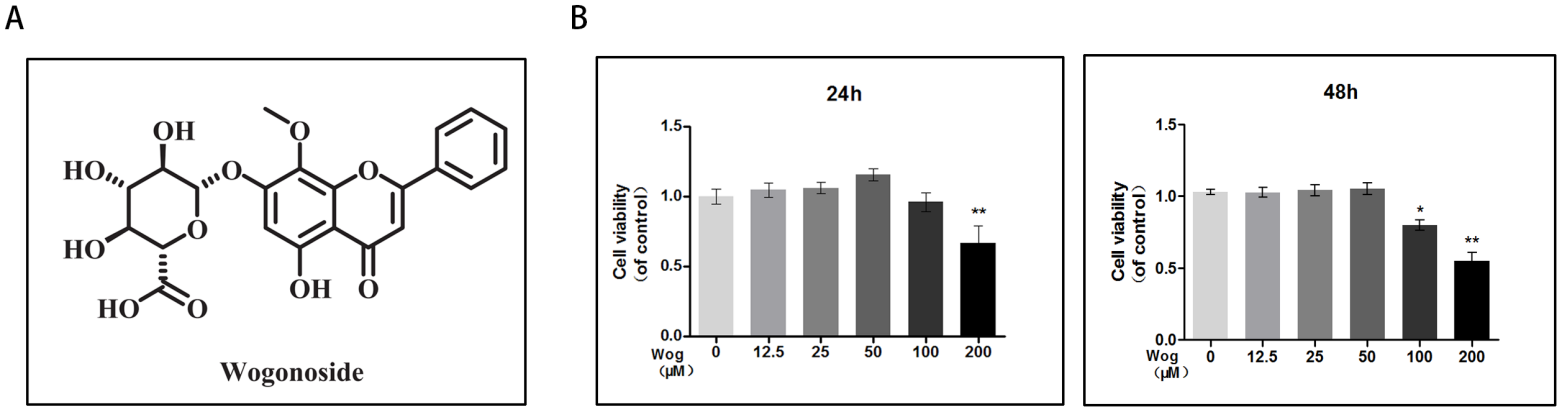
Effects of wogonoside on the cell viability of chondrocytes **(A)** Chemical structure of wogonoside. **(B)** The cytotoxic effect of wogonoside on chondrocytes was determined at various concentrations for 24 and 48 hours using a CCK8 assay. The values presented are the means ± S.D. of three independent experiments. *P < 0.05, **P < 0.01 vs. control group.

The cytotoxic effects of wogonoside on chondrocytes was determined at various concentrations (12.5, 25, 50, 100, and 200 μM) for 24 and 48 h using the Cell Counting Kit-8 (CCK8) assay. As shown in Figure 2B, treatment with 200 μM or 100 μM wogonoside significantly reduced cell viability at 24 h (**P<0.01 vs. untreated cells) and 48 h (*P<0.05 vs. untreated cells), respectively, indicating that cell viability is not affected by wogonoside at concentrations up to 50 μM before 48 h.Therefore, concentrations of 12.5, 25 and 50 μM wogonoside were used for subsequent experiments.

### Effects of wogonoside on the expression of iNOS, COX-2, PGE_2_, NO, TNF-α, and IL-6 in IL-1β-induced mouse chondrocytes

**Figure 3:**
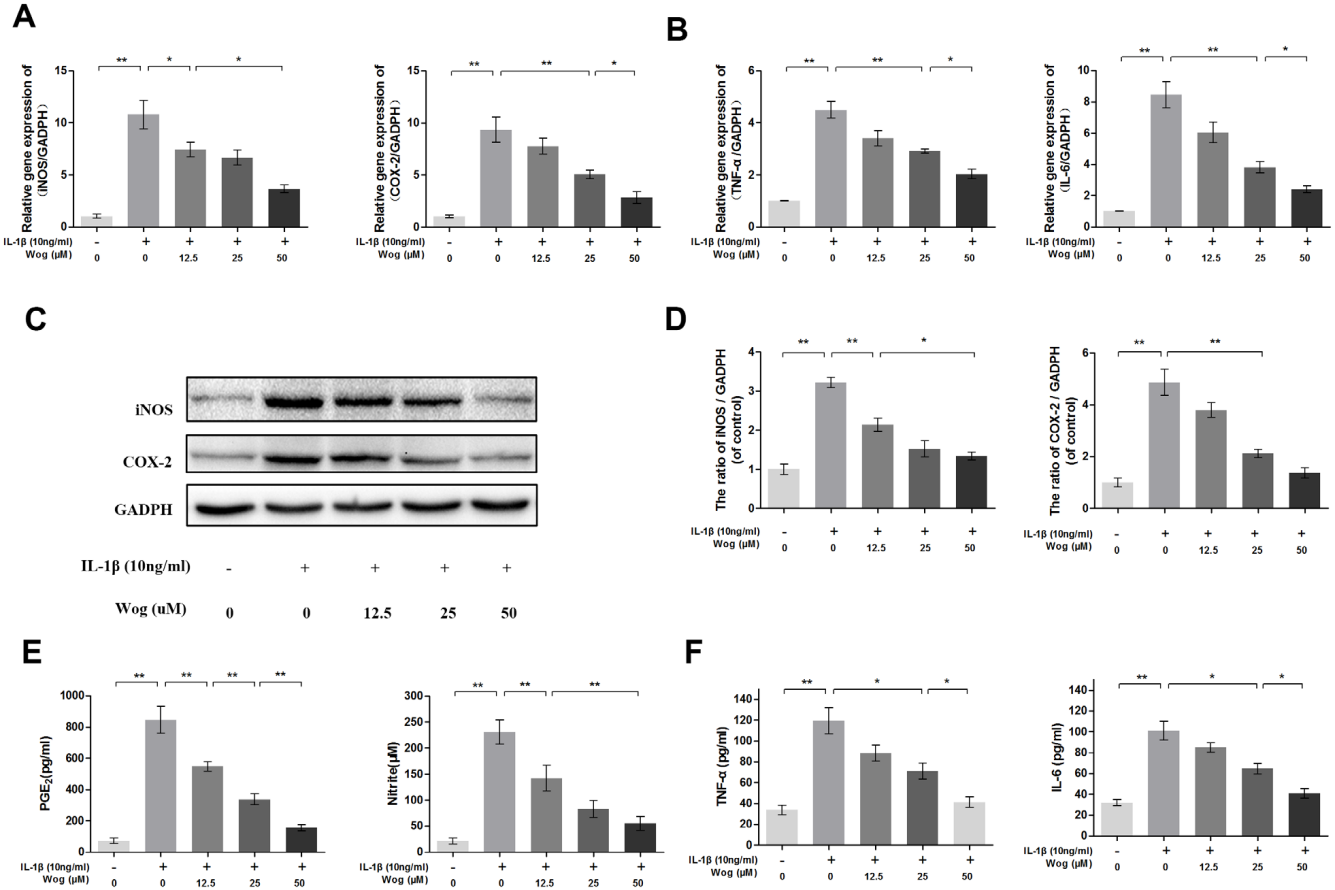
Wogonoside inhibit inflammatory effect in mice chondrocyte **(A-B)** The mRNA expression of iNOS, COX-2, TNF-α and IL-6 were measured by real-time PCR. **(C-D)** The protein expression of iNOS and COX-2 in chondrocytes treated as above. **(E-F)** Effect of wogonoside on IL-1β-induced PGE_2_, NO, TNF-α and IL-6 production in mice chondrocytes. The data in the figures represent the averages ± S.D. Significant differences between the treatment and control groups are indicated as **P<0.01, *P<0.05, n = 3.

We next determined whether wogonoside inhibits iNOS and COX-2 production at the transcriptional and/or translational level using real-time polymerase chain reaction (RT-PCR) and Western blot analysis. As shown in Figure 3A, 3C and 3D, wogonoside significantly inhibited the up-regulation of IL-1β (10 ng/ml)-induced mRNA and protein expression of iNOS and COX-2 in a dose-dependent manner (12.5, 25 and 50 μM). Moreover, the production of endogenous NO and PGE_**2**_ was significantly up-regulated by IL-1β stimulation. However, treatment with wogonoside resulted in decreased NO generation and PGE_2_ expression in a dose-dependent manner. In addition, a concentration–dependent inhibition of TNF-α and IL-6 production was observed by RT-PCR and enzyme-linked immunosorbent assay (ELISA) (Figure 3B and 3F). At the concentration of 12.5 μM, wogonoside inhibited the mRNA expressions of IL-6 and TNF-α but have no inhibitory effect on protein expressions. At the concentrations of 25 and 50 μM, wogonoside significantly inhibited TNF-α and IL-6 generation at both protein and mRNA levels. It suggests that wogonoside inhibits the production of these inflammatory cytokines at both gene and protein levels in a dose-dependent manner.

### Wogonoside protects against IL-1β-induced hypertrophic conversion and VEGF generation in mouse chondrocytes

**Figure 4:**
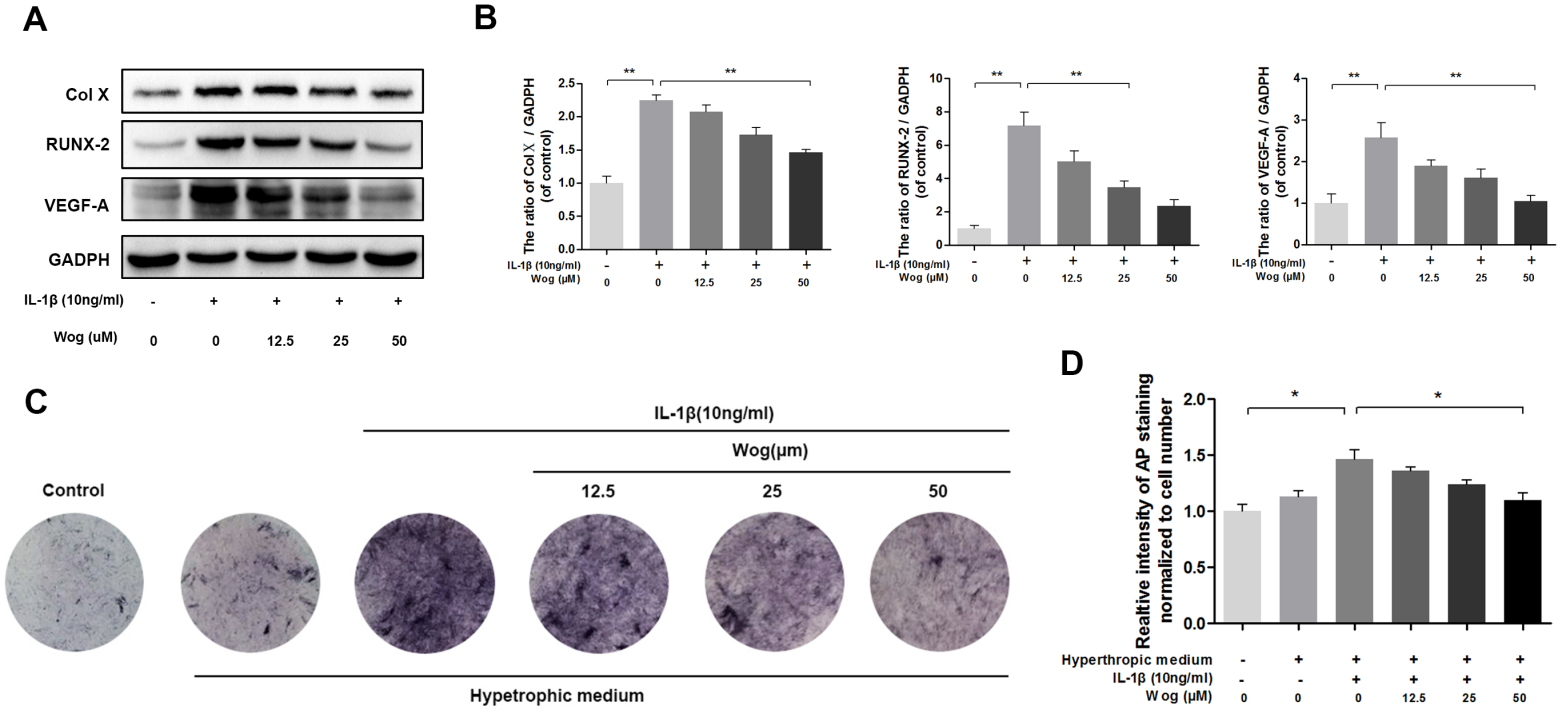
Wogonoside inhibit hypertrophic conversion and VEGF generation in mice chondrocyte **(A-B)** The protein expression of Col X, RUNX-2, and VEGF-A in chondrocytes treated as above. **(C-D)** wogonoside ameliorate IL-1β–induced chondrocyte hypertrophy and mineralization (as assessed by AP staining). The data in the figures represent the averages ± S.D. Significant differences between the treatment and control groups are indicated as **P<0.01, *P<0.05, n = 3.

The expression of collagen X and Runx-2, which are considered markers of chondrocyte hypertrophy, was examined by Western blotting, Both collagen X and Runx-2 were up-regulated in the presence of IL-1β, and wogonoside protected chondrocytes against hypertrophic conversion at concentrations of 25 μM (*P<0.05) and 50 μM (Figure 4A and 4B, **P<0.01). Alkaline phosphatase (ALP) activity is another indicator of hypertrophy and mineralization *in vitro*. Treatment with wogonoside resulted in an inhibition of ALP activity in a dose-dependent manner and reached a statistically significant difference at a concentration of 50 μM compared to other concentrations (Figure 4C and 4D, *P<0.05). Conversely, IL-1β led to the generation of VEGF, which is associated with the OA process. Pretreatment with wogonoside markedly attenuated the increase in VEGF compared to treatment with IL-1β alone at concentrations of 25 μM (*P<0.05) and 50 μM (**P<0.01).

### Effects of wogonoside on ECM synthesis in IL-1β-induced mouse chondrocytes

**Figure 5:**
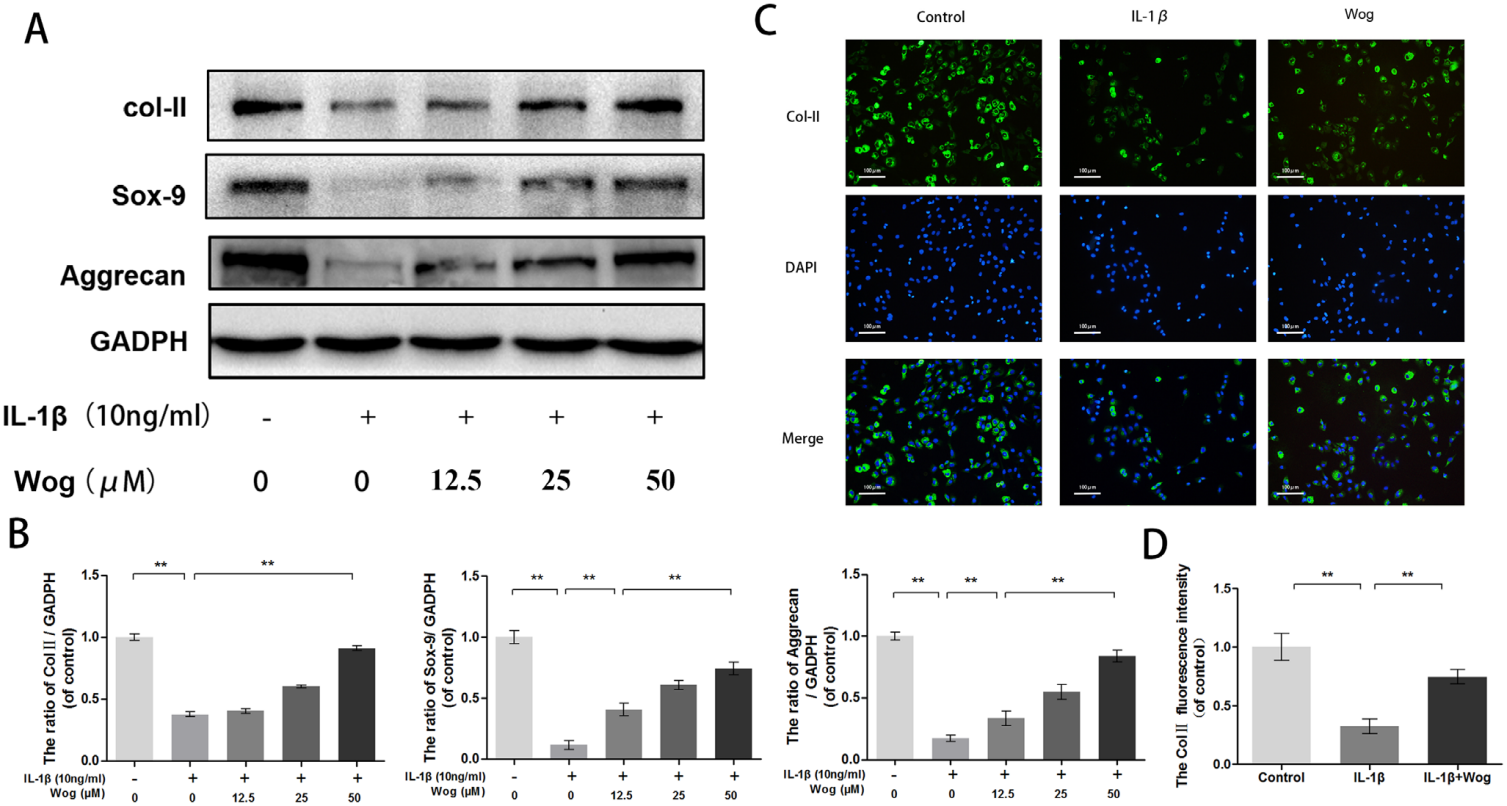
Wogonoside promote extracellular matrix synthesis from IL-1β induced mice chondrocyte **(A-B)** The protein expression of Col II, Sox-9, and aggrecan in chondrocytes treated as above. **(C-D)** The representative collagen II was detected by the immunofluorescence combined with DAPI staining for nuclei (original magnification × 100, scale bar: 100 μm). The data in the figures represent the averages ± S.D. Significant differences between the treatment and control groups are indicated as **P<0.01, *P<0.05, n = 3.

To evaluate chondrocyte degeneration, we investigated the function of ECM replacement of chondrocytes under IL-1β stimulation with or without wogonoside pretreatment by Western blot analysis. As shown in Figure 5A and 5B, IL-1β significantly inhibited Sox-9 transcription and decreased type II collagen and aggrecan synthesis (all **P<0.01). All alterations in IL-1β expression were reversed by pretreatment with wogonoside, especially at a concentration of 50 μM (**P<0.01). In addition, immunofluorescence showed that type II collagen-positive proteins mainly localized in the cytoplasm, consistent with the Western blot analysis (Figure 5C and 5D).

### Effects of wogonoside on ECM degradation in IL-1β-induced mouse chondrocytes

**Figure 6:**
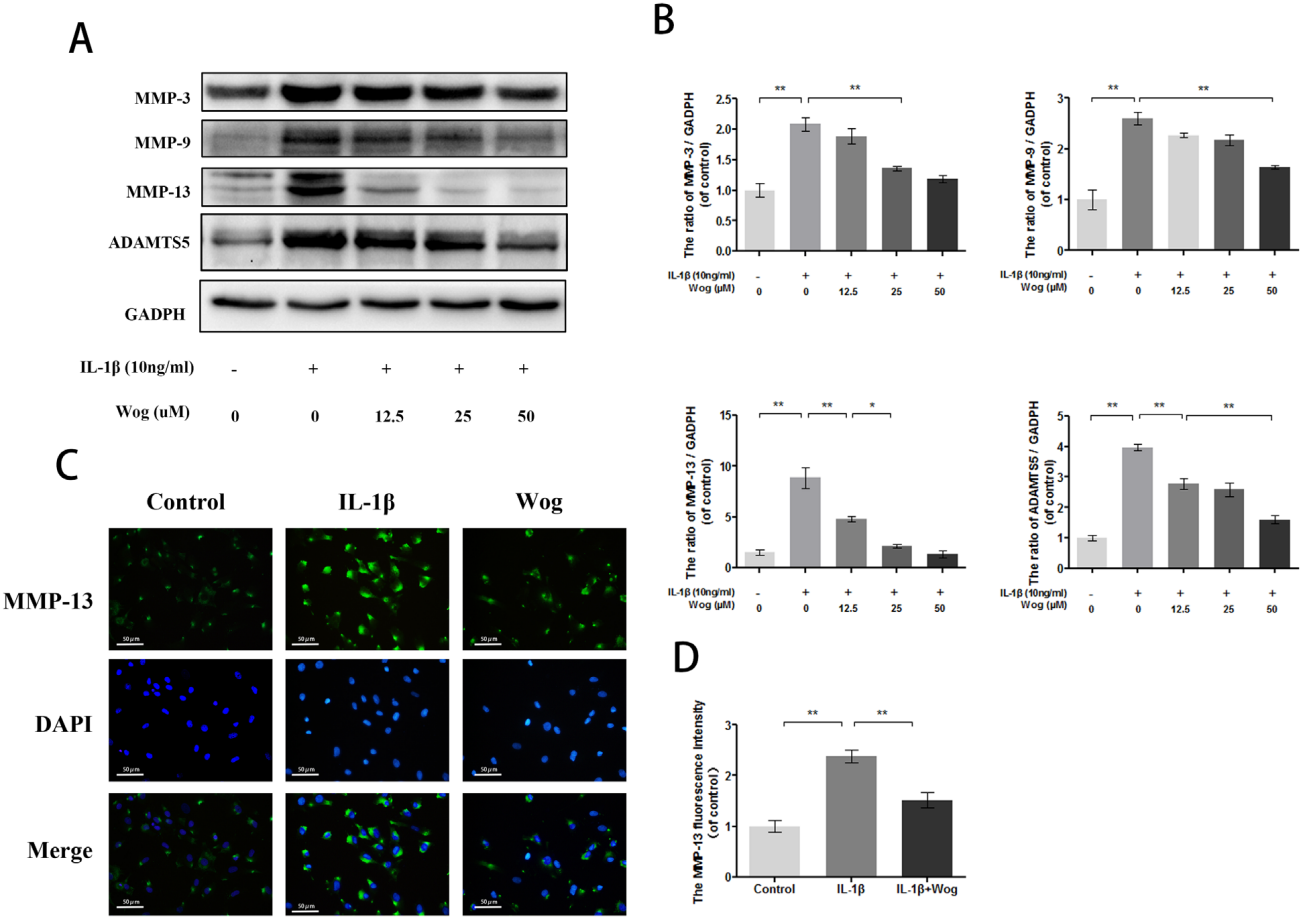
Wogonoside inhibit extracellular matrix degradation from IL-1β induced mice chondrocyte **(A-B)** The protein expression of MMP-3, MMP-9, MMP-13 and ADAMTS-5 in chondrocytes treated as above. **(C-D)** The representative MMP-13 was detected by the immunofluorescence combined with DAPI staining for nuclei (original magnification × 200, scale bar: 50 μm). The data in the figures represent the averages ± S.D. Significant differences between the treatment and control groups are indicated as **P<0.01, *P<0.05, n = 3.

MMPs and ADAMTS are regarded as major factors of ECM degradation. Western blot analysis showed that IL-1β significantly up-regulated the expression of MMPs such as MMP-3, MMP-9 and especially MMP-13 (Figure 6A and 6B, all **P<0.01). However, wogonoside reversed this destructive effect in a dose-dependent manner. Moreover, immunofluorescence staining of MMP-13 was consistent with the Western blot analysis. Similar results were obtained for ADAMTS-5 (Figure 6C and 6D).

### Time-dependent effects of IL-1β on the phosphorylation of IKK/IκBα/p65 and HIF-2α expression

**Figure 7:**
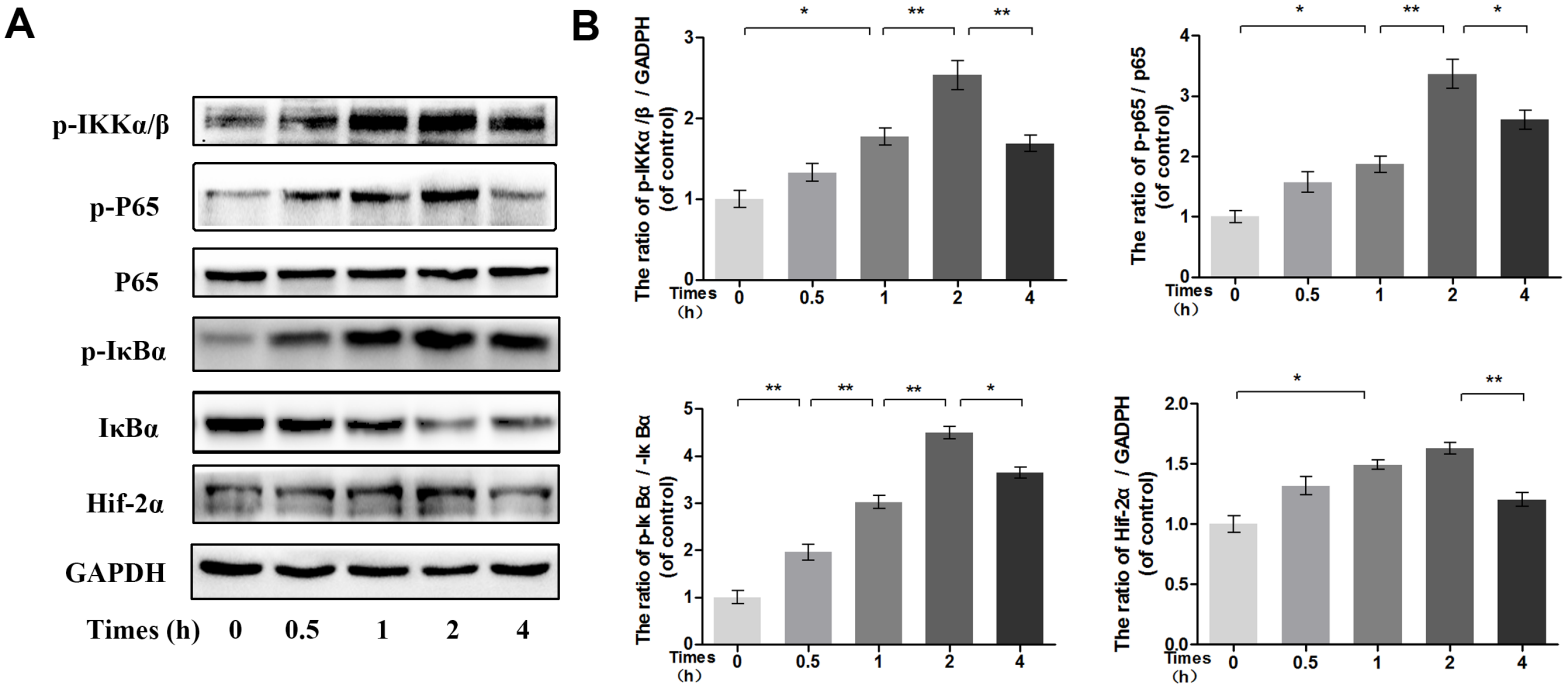
Time-dependent effect of IL-1β on phosphorylation of IKK/IκBα/p65 and HIF-2α expression **(A-B)** The protein expression of p-IKKα/β, p-IκBα, IκBα, p-p65, p65 and HIF-2α in chondrocytes treated as above. The data in the figures represent the averages ± S.D. Significant differences between the treatment and control groups are indicated as **P<0.01, *P<0.05, n = 3.

To determine the time at which NF-κB is phosphorylated, the phosphorylation levels of p65, IκBα and IKKα/β were assessed in IL-1β-stimulated chondrocytes by Western blot at the indicated time points. Western blot analysis at four time points revealed that IL-1β markedly induced the phosphorylation of IKKα/β, IκBα and NF-κB p65 in a time-dependent manner, with the phosphorylation response reaching a peak at approximately 2 h and decreasing rapidly at 4 h. Moreover, the expression of HIF-2α after IL-1β stimulation was consistent with the changes in the NF-κB pathway (Figure 7).

### Effects of wogonoside on IL-1β-induced NF-κB/HIF-2α activation

**Figure 8:**
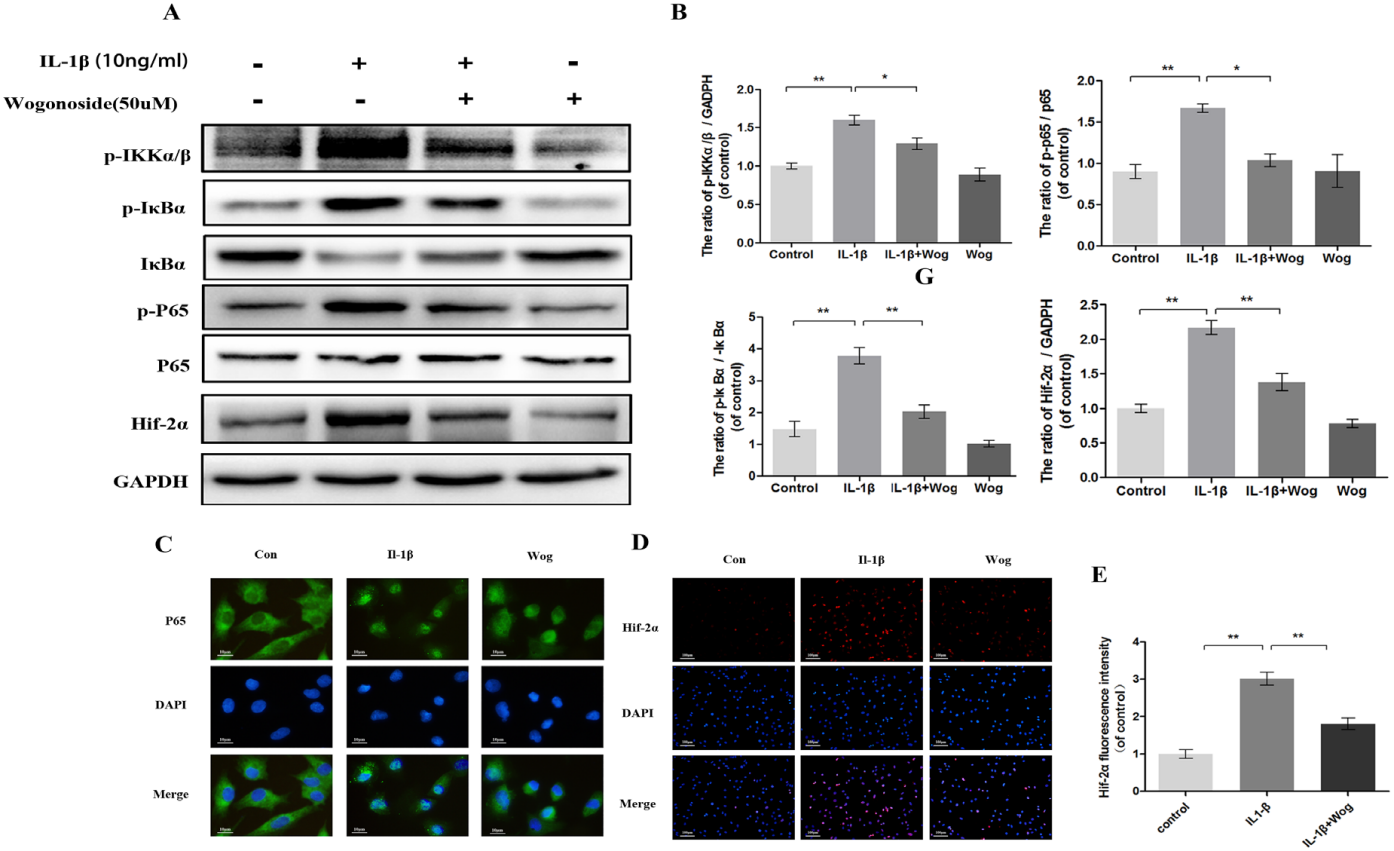
Effect of wogonoside on IL-1β-induced NF-κB/HIF-2α activation **(A-B)** The protein expression of p-IKKα/β, p-IκBα, IκBα, p-p65, p65 and HIF-2α in chondrocytes treated as above. **(C)** The nuclei translocation of p65 was etected by the immunofluorescence combined with DAPI staining for nuclei (original magnification × 400, scale bar: 10 μm). **(D-E)** The representative HIF-2α was detected by the immunofluorescence combined with DAPI staining for nuclei (original magnification × 100, scale bar: 100 μm). The data in the figures represent the averages ± S.D. Significant differences between the treatment and control groups are indicated as **P<0.01, *P<0.05, n = 3.

To examine the effects of wogonoside on NF-κB pathway activation, the phosphorylation levels of p65, IκBα and IKKα/β and the expression of HIF-2α were assessed in IL-1β-stimulated chondrocytes by Western blot analysis at 2 h. IL-1β markedly induced the phosphorylation of IKKα/β, IκBα and p65 and up-regulated HIF-2α expression (Figure 8A and 8B, all **P<0.01). However, this response was significantly decreased by wogonoside pretreatment at a concentration of 50 μM (Figure 8A and 8B, all *P<0.01).

Indirect immunofluorescence staining of p65 was performed to reveal the translocation of p65 from the cytoplasm to the nucleus in chondrocytes in response to NF-κB activation by IL-1β. The administration of chondrocytes were divided into three groups: (1) control group; (2) IL-1β stimulated group; (3) IL-1β plus wogonoside treatment group. In the control group, the majority of p65 protein was located in the cytoplasm. Upon IL-1β stimulation, p65-positive proteins stained intensively in the nucleus of chondrocytes, leaving the cytoplasm unstained, indicating the nuclear translocation of the NF-κB subunit. However, pretreatment with wogonoside inhibited the translocation of p65 subunits into the nucleus (Figure 8C). These immunomorphological findings suggest an inhibitory effect of wogonoside on the IL-1β-induced p65 nuclear dislocation in chondrocytes and are consistent with the inhibition of the NF-κB pathway observed by Western blot.

HIF-2α expression was also detected by indirect immunofluorescence staining (Figure 8D and 8E). The results indicated that a considerable amount of HIF-2α is localized to the nucleus upon IL-1β stimulation for 2 h compared to control chondrocytes. Fluorescence intensity was inhibited by wogonoside pretreatment for 24 h, indicating that the protective effects of wogonoside are associated with HIF-2α inhibition.

### Effects of wogonoside on IL-1β-induced activation of Stat3, ERK1/2, TRAF6, IRAK1 and PI3K/AKT

**Figure 9:**
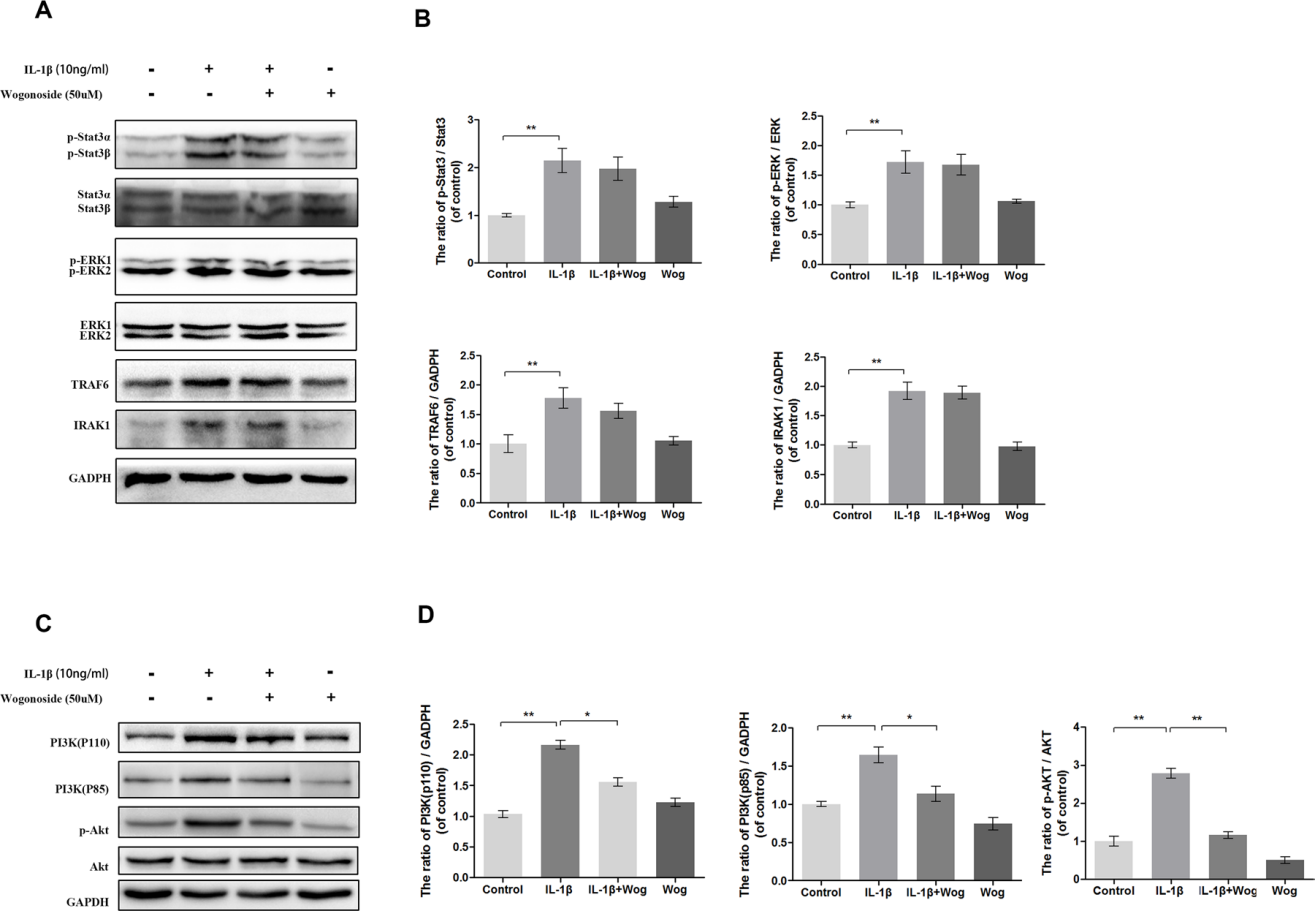
Effect of wogonoside on IL-1β-induced PI3K/AKT activiation **(A-B)** The protein expression of p-Stat3, Stat3, p-ERK1/2, ERK1/2, TRAF6 and IRAK1 in chondrocytes treated as above. **(C-D)** The protein expression of PI3K(p110), PI3K(p85), p-AKT and AKT in chondrocytes treated as above. The data in the figures represent the averages ± S.D. Significant differences between the treatment and control groups are indicated as **P<0.01, *P<0.05, n = 3.

To clarify further the anti-inflammatory mechanism of wogonoside, several upstream signaling molecules associated with IL-1β induced inflammation in chondrocytes were be examined, such as Signal Transducer and Activator of Transcription (Stat3), Extracellular signal-regulated kinase 1/2 (ERK1/2), TNF Receptor Associated Factor (TRAF6), Interleukin-1 Receptor Associated Kinase (IRAK1) and PI3K/AKT. As shown in Figure 9, all of these signaling molecules were activated with IL-1β treatment. Although pretreated with wogonoside did not decrease the protein expressions of stat3, ERK1/2, TRAF6 and IRAK1, the catalytic subunit (PI3K-p110) and the regulatory subunit of PI3K (PI3K-p85) were significantly down-regulated by wogonoside in IL-1β-stimulated chondrocytes according to results of western blot analysis. IL-1β-induced AKT phosphorylation was also inhibited by wogonoside.

### Pharmacokinetic profle of wogonoside

**Figure 10:**
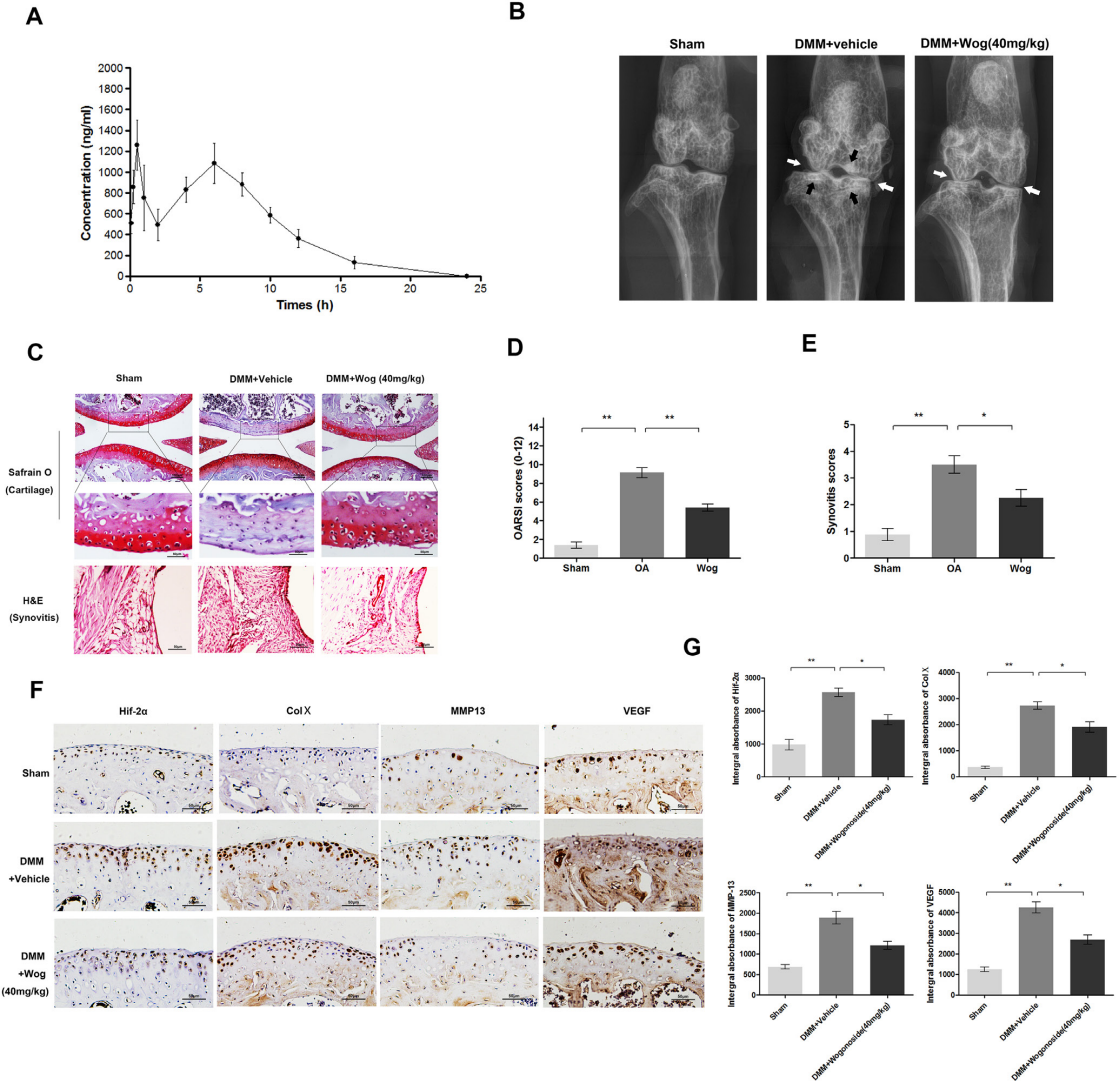
Wogonoside ameliorates OA development in mice DMM model *in vivo* **(A)** Concentration-time curve of wogonoside administration in rats. **(B)** Digital X-ray image of mouse knee joints from different experimental groups. Narrowing of joint space was found in both OA and treatment group (white arrows), the calcification of cartilage surface was obviously shown in OA group (black arrows). **(C)** Representative S-O staining of cartilage and H&E staining of synovitis from different experimental groups at 8 weeks post-surgery (original magnification × 100 or ×400, scale bar: 100 μm or 50μm). **(D)** Diagrams showed the OARIS socres of cartilage. **(E)** Diagrams showed the scores of synovitis. **(F-G)** immunohistochemical staining of HIF-2α, Col X, MMP-13 and VEGF-A expression in the cartilage samples (original magnification × 100, scale bar: 50 μm). The data in the figures represent the averages ± S.D. Significant differences between the treatment and control groups are indicated as **P<0.01, *P<0.05, n = 3.

The pharmacokinetic profle of wogonoside at doses of 20 mg/kg in rats (equal 40mg/kg in mice) are presented in (Figure 10A). Each point represents a mean value of six serum concentrations. After intragastric administration, wogonoside was quickly absorbed into the bloodstream and reached first peak serum concentration (C_max_1) of 1378.25±105.13 ng/ml at 0.54±0.25 h (T_max_1). The second peak was reached at 5.67±0.82 h (T_max_2) with the second peak serum concentration (C_max_ 2) of 1131.07±127.94 ng/ml. Additionally, the area under the serum concentration-time curve (AUC_0-24_) at 24 h is 10655.58±693.41 h*ng/ml.

### Wogonoside ameliorates OA development in the mouse model of DMM

To investigate the protective effects of wogonoside in OA development *in vivo*, a surgically induced mouse model of DMM was established, followed by intragastric administration of 40 mg/kg wogonoside dissolved in 0.5% Carboxymethylcellulose (CMC) or vehicle (0.5% CMC alone) once daily for 8 weeks. Histological analysis of OA was evaluated by Safranin O staining for cartilage, and hematoxylin and eosin (H&E) staining for synovitis. Osteoarthritis Research Society International (OARSI) scores and synovitis scores were used for the quantitative analysis. As shown in the Safranin O staining (Figure 10C), the cartilage surface was smooth and showed positive red staining in the sham control group. The OA group showed cartilage erosion, apparent hypocellularity and massive proteoglycan loss compared to the sham control group. However, the wogonoside group exhibited less proteoglycan loss compared to the OA group. Consistent with the Safranin O staining, the OARSI scores (Figure 10D) of the OA group were markedly higher (9.13 ± 1.46) than those of the sham control (1.25 ± 0.20). In contrast, the wogonoside group displayed clearly lower OARSI scores (5.50 ± 1.06) than the OA group. In addition, the synovial thickening and hypercellularity was presented in OA group, whereas, wogonoside treatment alleviated synovitis compared to the OA group (Figure 10E). What’s more, according to the X-ray shown, the OA group showed severe knee varus, aberrant narrowing of joint space and increase of cartilage surface density compared to the sham group. Nevertheless, although the narrowing of joint space exits, the calcification of cartilage surface was milder in wogonoside treatment group (Figure 10B). Together, these results indicate that wogonoside protects against OA development *in vivo* via inhibiting the loss of proteoglycan, cartilage calcification and synovitis.

### Wogonoside attenuates ECM degradation and hypertrophy of chondrocytes *in vivo* via HIF-2α inhibition

To demonstrate the mechanism of the chondroprotective effects of wogonoside *in vivo*, immunohistochemical staining of HIF-2α, MMP-13, collagen X, and VEGF was performed. HIF-2α-, MMP-13-, collagen X-, and VEGF-positive areas that were elevated in the cartilage of the OA group compared to the sham group were markedly reversed after wogonoside administration (Figure 10F and 10G).

## DISCUSSION

The main finding of this study is that wogonoside can reverse IL-1β-induced PI3K/AKT activation and nuclear dislocation of NF-κB in mouse chondrocytes, which subsequently protect against ECM degradation and hypertrophic conversion in the presence of IL-1β. In addition, the *in vivo* results suggest that wogonoside may play a protective role in OA via HIF-2α inhibition.

With the rapid aging of the population, the incidence rates of OA have been continuously increasing in recent years [28]. However, the pathogenesis of OA development is not clear and no effective intervention exists to prevent the progression of OA unless total knee arthroplasty is performed at the end stage of OA [29, 30]. Although non-steroidal anti-inflammatory drugs (NSAIDs) have been widely used for OA treatment in clinical practice during the last few years, these agents only relieve the clinical symptoms temporarily and fail to prevent the progression of OA; they also have many side effects [22]. Therefore, it is necessary to identify novel chemopreventive agents that can reverse cartilage destruction during the progression of OA with minimal or no side effects or toxicity.

Wogonoside, a glucuronide metabolite of the bioactive flavonoid wogonin, exhibits anti-inflammatory, anti-viral, anti-oxidant, and anti-cancer properties [31]. Previous studies have reported the anti-inflammatory effects of wogonoside in several diseases [25–27]. For the first time, we investigated its anti-inflammatory effects in chondrocytes. Furthermore, several *in vitro* and *in vivo* studies have confirmed that the inflammatory environment plays a negative role in the stability of chondrocyte phenotype and hemostasis of the ECM, which aggravates cartilage destruction and promotes OA progression [8–10]. Therefore, we further investigated whether the administration of wogonoside can reverse these conditions caused by inflammation and explored the potential molecular mechanisms involved.

One of the most apparent phenomena in the inflammatory environment in chondrocytes is the increase in NO, a reactive molecule originating from the guanidine nitrogen of L-arginine, which is catalyzed by iNOS [32]. NO suppresses collagen II and proteoglycan synthesis and stimulates the activation of MMPs to cause ECM degradation [33]. Conversely, PGE_2_ is a principal mediator of inflammation that is converted from IL-1β-induced endogenous arachidonic acid via the COX-2 catalytic reactions and is associated with cartilage destruction due to its powerful activation on MMPs and other inflammatory mediators [34, 35]. All of these factors, as well as TNF-α and IL-6, are important inflammatory cytokines in OA development [36]. In this study, we found that the over-production of PGE_2_ and NO, as well as the up-regulation of COX-2 and iNOS, at both the gene and protein levels are inhibited by wogonoside following IL-1β stimulation. Similar results were also found for TNF-α and IL-6. These results are consistent with those from Yang et al., whose research revealed that wogonoside inhibits LPS-induced inflammatory activation in RAW264.7 macrophages [26]. Therefore, we surmised that the anti-inflammatory effects of wogonoside may be associated with the inhibition of TNF-α and IL-6 expression, as well as the decreased production of NO and PGE_2_ via targeting iNOS and COX-2.

A stable phenotype is essential for ECM replacement in normal hyaline cartilage in chondrocytes. However, chronic stimulation of inflammatory cytokines can lead to an unrestricted hypertrophic shift. This aberrant terminal differentiation results in excessive calcification of chondrocytes in OA development [8]. Type X collagen, MMP-13 and RUNX-2 are major markers of hypertrophic chondrocytes, which are markedly elevated following IL-1β stimulation, as shown in our study, and are reduced by wogonoside pretreatment. Similar changes were observed regarding VEGF, which is also related to chondrocyte hypertrophy. However, the main function of VEGF is associated with angiogenesis, which is crucial for OA progression according to recent research [37]. Moreover, the up-regulation of ALP activity, a marker of the calcification of chondrocytes, represents the terminal change of hypertrophic chondrocytes. Our study revealed that AP staining was consistent with the Western blot analysis of hypertrophy-related proteins, indicating that wogonoside attenuates the increase in ALP activity from IL-β-induced chondrocytes.

The ECM secreted by chondrocytes mediates the principle function of cartilage. Sox-9 is the core gene for type II collagen synthesis [7]. In contrast, MMPs and ADAMTS contribute to ECM catabolism [11–13]. Protecting ECM hemostasis is the pivotal and ultimate goal for most interventions in OA treatment [6]. According to our research, in the presence of IL-β, wogonoside not only enhanced Sox-9 transcription and promoted synthesis of the most prominent components of the ECM, including type II collagen and aggrecan, but also inhibited the catabolism of ECM components by down-regulating MMPs and ADAMTS-5, which helped maintain ECM homeostasis.

To elucidate the mechanism by which wogonoside unites anti-inflammation, anti-hypertrophy and anti-catabolism of the ECM in chondrocytes, we further explored the cross-talk between the PI3K/AKT, NF-κB and HIF-2α signaling pathways. NF-κB plays a key regulatory role in inflammatory mediators involved in the pathogenesis of OA [38]. According to previous studies, IL-1β stimulation triggers the phosphorylation of the catalytic subunits of IKK (IKKα and IKKβ), as well as the binding protein of p65 (IκBα), which consequently frees p65 and translocates it from the cytosol to the nucleus and leads to the production of catabolic enzymes, cytokines and inflammatory mediators [16]. Furthermore, several studies have demonstrated that NF-κB activation is associated with downstream regulators of terminal chondrocyte differentiation, including HIF-2α and Runx2. Additionally, as shown in our study, the up-regulation of HIF-2α expression was followed by NF-κB activation, both of which were affected in a time-dependent manner. It was consistent with the studies of Chun et al [19] and Kawaguchi et al [20], indicating that HIF-2α is located downstream of the NF-κB pathway, which relates inflammation with the phenotypic and functional changes in IL-1β-induced chondrocytes. For the first time, our data reveal that wogonoside inhibits IL-1β-induced inflammation, ECM degradation and hypertrophy by suppressing NF-κB/HIF-2α signaling in chondrocytes. Additionally, wogonoside has been reported to inhibit the inflammatory effects in macrophages and tumor cells via NF-κB inhibition [25, 39]. Thus, the results from previous studies along with ours indicate that the anti-inflammatory effects of wogonoside may be closely related to the inhibition of NF-κB activation. The benefits of phenotypic and functional alterations may also be attributed to the vertical inhibition of HIF-2α.

Several upstream signaling molecules, such as Stat3, ERK1/2, TRAF6, IRAK1 and PI3K/AKT were reported to be involved in IL-1β induced inflammation in chondrocytes. In our studies, all of these protein expressions were examined. The protein expression of Stat3, ERK1/2, TRAF6 and IRAK1 were not altered after wogonoside administration with or without IL-1β stimulation. However the activation of PI3K/AKT after IL-1β stimulation was inhibit by wogonoside (Figure 9). PI3K/AKT is one of the most well-studied upstream signaling pathways of intracellular message trafficking. It regulates a cascade of changes through its broad target proteins such as mechanistic target of rapamycin (mTOR), NF-κB, glycogen synthase kinase 3 beta (GSK-3β), and p53, all of which are involved in the OA process [17]. Therefore, we further investigated the correlation between NF-κB and PI3K/AKT signaling during wogonoside treatment with respect to OA. The PI3K family consists of three classes, Class I, Class II and Class III, among which the Class I PI3Ks are responsible for the production of PIP3, which binds to the pleckstrin homology domain of AKT and phosphoinoside-dependent protein kinase 1 (PDK1), resulting in the phosphorylation of AKT [40–42]. In the current study, IL-1β stimulation markedly up-regulated the protein levels of the catalytic subunit (PI3K-p110) and the regulatory subunit (PI3K-p85) of PI3K, which catalyzes the production of PIP3 and leads to the phosphorylation of AKT followed by activation of NF-κB. Our data revealed that wogonoside blocks IL-1β-induced AKT activation via inhibition of PI3K-p110 and PI3K-p85, which further targets the downstream NF-κB/HIF-2α molecules.

In order to examine the effects of wogonoside *in vivo*, a pharmacokinetic study of wogonoside was conducted. Due to the limited blood volume in mice, the rats were used into our studies through intragastric administration. The dosage to rats was converted by using body surface area normalized methods [48] The results of time-concentration curve showed a double peaks phenomena after administration. The peak serum concentration (Cmax) of 1825.4 and 6635.7 nM at 0.5 h and 8 h respectively post-dose for dose 20mg/kg. This could be due to enterohepatic circulation, which might prolong the effective duration of agents. The area under the curve (AUC_0-24_) at 24 h is 10655.58±693.41 h*ng/ml. All of these data indicated that the oral administration of wogonoside at 20mg/kg once a day in rat (equally 40mg/kg in mice) showed a sufficient systemic exposure and good dose proportionality for pharmacodynamics evaluation. And it is an important property for wogonoside in its translational development from animal to human subjects.

The loss of proteoglycans in the cartilage surface, hypertrophic changes, degradation of the ECM, angiogenesis, and synovitis are involved in OA progression [8]. Among them, the decrease in proteoglycans is the main characteristic of cartilage degeneration. Hypertrophic changes and degradation of the ECM also play key roles in OA development. Angiogenesis causes vessels to invade the hyaline cartilage and results in the calcification of cartilage, which is believed to contribute to the pathogenesis of OA [37]. Moreover, synovitis is closely related to clinical symptoms such as joint dysfunction and may aggravate cartilage degeneration [43]. DMM was demonstrated to be a reliable and effective method to establish an animal OA model for *in vivo* analysis [44]. Thus, in the present study, mouse OA models induced by DMM were used to examine the chondroprotective effects of wogonoside *in vivo*. Treatment with wogonoside dramatically reduced the OARSI scores as well as the scores of severity of synovitis in mouse OA models. Conversely, wogonoside inhibited MMP-13, type X collagen and VEGF generation by surgical intervention via HIF-2α inhibition, indicating that HIF-2α is a potential target of wogonoside that could be used for OA treatment.

**Figure 11:**
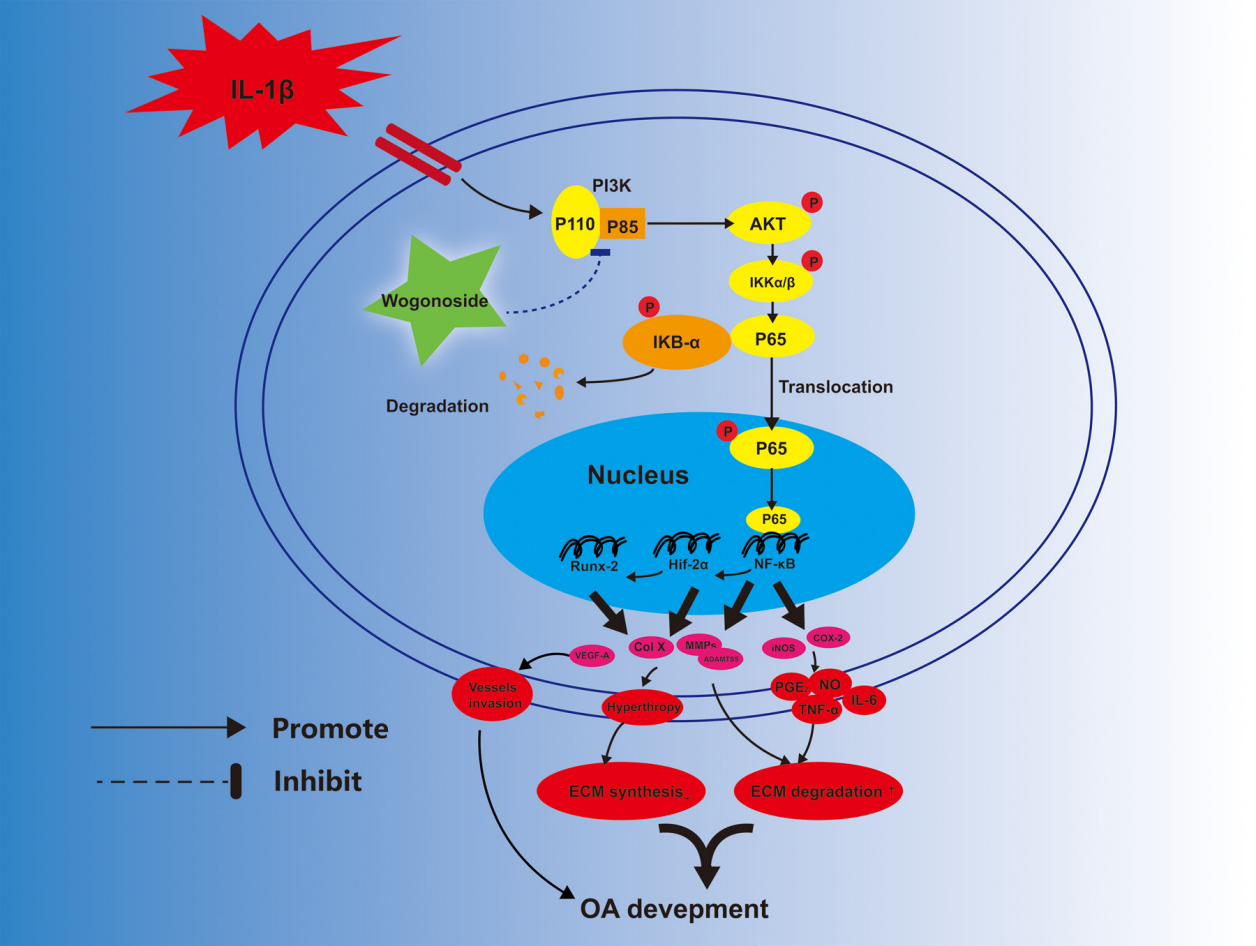
Potential molecular mechanism involved in wogonoside treatment in chondrocytes Wogonoside attenuated IL-1β induced ECM degradation and hypertrophy in mice chondrocyte via suppressing NF-Κb/ HIF-2α activation by PI3K/Akt pathway.

In conclusion, the current study demonstrates that wogonoside prevents IL-1β-associated ECM degradation and hypertrophic conversion in mouse chondrocytes. The potential mechanism involved in its protective effects is the reversal of inflammation-related destruction via inhibiting NF-κB/HIF-2α activation through the PI3K/AKT pathway (Figure 11). Moreover, oral administration of wogonoside not only reduced cartilage degradation, hypertrophic conversion and angiogenesis but also relieved synovitis in mouse OA models via HIF-2α inhibition. Therefore, wogonoside may serve as a promising effective chemotherapeutic agent for the treatment of OA in the future.

## MATERIALS AND METHODS

### Ethics statement

All surgical interventions, treatments and postoperative animal care procedures were performed in strict accordance with the Animal Care and Use Committee of Wenzhou Medical University (wydw2014-0129).

### Reagents and antibodies

Wogonoside (purity >98 %), was purchase from Nantong Feiyu Biological Technology Co, ltd (Nantong, China). Carboxymethylcellulose (CMC) and type II collagenases were purchased from Sigma-Aldrich (St Louis, MO, USA). Recombinant human IL-1β was purchased from PeproTech (NJ, USA). The primary antibody against collagen II, aggrecan, MMP-9, MMP-13, ADAMTS5, collagen X, HIF-2α, TRAF6, p-ERK1/2, ERK1/2, p-Stat3, Stat3 and GADPH were acquired from Abcam (Cambridge, UK), MMP-3 and iNOS antibodies were obtained from Sigma-Aldrich (St Louis, MO, USA). Sox-9 antibody was obtained from Santa Cruz Biotechnology (Santa Cruz, CA, USA); Anti-VEGF-A, Anti-Runx-2, Anti-IRAK1, goat anti-rabbit, and anti-mouse IgG-HRP was from Bioworld (OH, USA) and antibodies against COX-2, PI3K(p110), PI3K(p85), AKT, p-AKT, p-IKKα/β, IκBα, p-IκBα, p65, and p-p65 were purchased from Cell Signaling Technology (Danvers, MA, USA); Alexa Fluor®488 labeled and Alexa Fluor®594 labeled Goat Anti-Rabbit IgG (H+L) second antibody was purchased from Jackson ImmunoResearch (West Grove, PA, USA). The 4', 6-diamidino-2-phenylindole (DAPI) was obtained from Beyotime (Shanghai, China). The cell culture reagents were purchased from Gibco (Grand Island, NY, USA).

### Primary mice chondrocytes culture

Ten immature C57BL/6 mice (5 males and 5 females, 10 days) were euthanized with an overdose of sodium pentobarbital. The knee cartilages of mice were collected carefully under aseptic conditions by a dissecting microscope, and the tissues were treated with 2mg/ml (0.1%) collagenase II for 4 h at 37 °C. Next, the digested cartilage tissues were suspended and seeded into tissue culture flasks. The chondrocytes grow in DMEM/F12 (Gibco, Invitrogen, Grand Island, NY) with 10% fetal bovine serum (FBS; Hyclone, Thermo Scientific, Logan, UT, USA) and 1% penicillin/streptomycin antibiotics (Gibco, Invitrogen, Grand Island, NY) in the incubator maintained at 5% CO_2_ at 37 °C. The medium was changed firstly after 24 h incubation. When up to 80% to 90% confluency, the cells were harvested by using 0.25% Trypsin-EDTA (Gibco, Invitrogen). Then, cells were replanted into 10 cm culture plates at the appropriate density. The second-passage chondrocytes were used for all of our experiment due to no significant changes was noticed during cells passaging from passage 0 to passage 2. The chondrocytes were cultured in the incubator maintained at 5% CO_2_ at 37 °C and the complete medium was changed every other day.

### Alcian blue staining

The phenotype of chondrocytes identified by staining for sulfate proteoglycan with Alcian blue as previously described [45]. The passage two chondrocytes were washed twice with cold PBS and fixed with 4% paraformaldehyde at room temperature for 15 min. After washed twice by PBS again, the chondrocyte will be pretreated with 0.1 M HCl for 3 min, Next, the cells were stained 30min with 0.1% Alcian Blue 8GX (Sigma-Aldrich, St Louis, MO, USA). After washing 3 times with distilled water, the stained cells were observed and captured observed with invert microscope (Leica).

### Cell viability assay

The cytotoxicity of wogonoside on chondrocytes were detected with the cell counting kit-8 (CCK-8; Dojindo Co, Kumamoto, Japan) according to the manufacturer’s protocol. Firsrly, the second-passage chondrocytes were transferred to 96-well plates (50000 cell/ cm^2^) and incubated in different concentration of wogonoside(12.5,25,50,100,200 μM) for 24 h. At the indicated time, the cells were washed with phosphate-buffered saline (PBS), and then 100 μl of DMEM/F12 containing 10μl of CCK-8 solution was added to each well of the plate and incubated for another 2 h at 37°C. The absorbance of the wells was then measured at 450 nm using a micro-plate reader. All experiments were performed triplicately.

### NO, PGE_2_, TNF-α, IL-6 measurement

The interaction of NO in culture medium was measured by Griess reagent as previously described [46]. The concentreation of PGE_2_, TNF-α and IL-6 in cell culture supernatants was detected by using commercial ELISA kits (R&D Systems, Minneapoils, MN) according to the manufacturer's instructions. All assays were performed in triplicate.

### Real-time PCR

The total RNA of chondrocytes stimulated with IL-1β(10ng/ml) and wogonoside at various concentrations were extracted from the cells in 6-cm culture plates using TRIzol reagent (Invitrogen). 1000ng of total RNA was reverse transcribed to synthesize cDNA (MBI Fermantas, Germany). For the quantitative realtime PCR (qPCR), a total 10 μl of reaction volume was used, including 5 μl of 2 × SYBR Master Mix, 0.25 μl of each primer and 4.5 μl of diluted cDNA. Parameters of RT-PCR were: 10 min 95 °C, followed by 40 cycles of 15 s 95 °C and 1 min 60 °C. The reaction was performed using CFX96Real-Time PCR System (Bio-Rad Laboratories, California, USA). The cycle threshold (Ct) values were collected and normalized to the level of GAPDH. The level of relative mRNA of each target gene was calculated by using the 2^-ΔΔCt^ method. The primers of COX-2, iNOS, IL-6, TNF-α were designed with the aid of NCBI Primer-Blast Tool. (https://www.ncbi.nlm.nih.gov/tools/primer-blast/), which were listed as follows: COX-2 (F) 5′- TCCTCACATCCCTGAGAACC-3′, (R) 5′- GTCGCACACTCTGTTGTGCT-3′; iNOS (F) 5′- GACGAGACGGATAGGCAGAG-3′, (R) 5′- CACATGCAAGGAAGGGAACT-3’; IL-6, (F) 5′-CCGGAGAGGAGACTTCACAG-3′, (R) 5′- TCCACGATTTCCCAGAGAAC-3′; TNF-α (F) 5′-ACGGCATGGATCTCAAAGAC-3′, (R) 5′- GTGGGTGAGGAGCACGTAGT- 3′.

### Western blotting

The total protein extracted from chondrocytes was isolated using RIPA lysis buffer with 1 mM PMSF (Phenylmethanesulfonyl fluoride) and on the ice for 10 min followed by 15 min centrifugation at 12000 rpm and 4°C, and then protein concentration was measured using the BCA protein assay kit (Beyotime). 40 ng of protein was separated by sodium dodecylsulfate-polyacrylamide gel electrophoresis (SDS PAGE) and transferred to a polyvinylidene difluoride membrane (Bio-Rad, USA). After blocking with 5% nonfat milk for 2 h, the membranes were incubated with the primary antibody against collagen II (1:1000), Aggrecan (1:1000), Sox9 (1:250), Col X(1:500), Runx-2(1:500), VEGF-A (1:500), GADPH (1:5000), iNOS (1:1000), COX-2 (1:1000), MMP-3 (1:1000), MMP-9 (1:1000), ADAMTS-5 (1:1000), p-IKKα/β (1:500), p-p65 (1:1000), p65 (1:1000), p-IκBα (1:1000), IκBα (1:1000), p-Stat3 (1:1000), Stat3 (1:1000), p-ERK1/2 (1:1000), ERK1/2 (1:1000), TRAF6 (1:1000), IRAK1 (1:1000), PI3K(p110) (1:1000), PI3K(p85) (1:1000), p-AKT (1:1000), AKT (1:1000) and HIF-2α (1:1000) overnight at 4 °C, and followed by subsequently incubation with respective secondary antibodies for 2h at room temperature. After 3 times washing with TBST, the blots were visualized by electrochemiluminescence plus reagent (Invitrogen). Finally, the intensity of these blots were quantified with Image Lab 3.0 software (Bio-Rad).

### Immunofluorescence

For collagen II and MMP-13 staining, the chondrocytes were planted in glass plates in a six-well plate and then the cells were treated with 10 ng/ml IL-1β or being co-treated with 10 ng/ml IL-1β and 50 μM wogonoside for 24 h in medium after incubated with serum-starved medium overnights. For p65 and HIF-2αstaining, the duration of IL-1β and wogonoside treatment was down to 2 h. After treatments. The samples were rinsed three-times in PBS before fixation using 4% paraformaldehyde and followed by permeation using the 0.1% Triton X-100 diluted in PBS for 15 min. Then the cells were blocked with 5% bovine serum albumin for 1 h at 37°C, rinsed with PBS and incubated with primary antibodies which diluted in PBS: collagen II (1:200), MMP-13 (1:200), p65 (1:200) and HIF-2α (1:400) in a humid chamber overnight at 4 °C. On the next day, the glass plates were washed and incubated with Alexa Fluor®488 labeled or Alexa Fluor®594 conjugated second antibodies (1:400) for 1 h at room temperature and labeled with DAPI for 5 min. Finally, three fields of each slides were chosen randomly for microscopic observation with a fluorescence microscope (Olympus Inc., Tokyo, Japan), and the fluorescence intensity was measured using Image J software 2.1 (Bethesda, MDUSA) by observers who were blinded to the experimental groups.

### *In vitro* chondrocyte hypertrophy and mineralization assay

The hypertrophy and mineralization of chondrocyte was determined by AP staining as previous described [47]. Cells were seeded into 24-well plates and incubated with differentiation medium for 14 days, which composed of DMEM/F12 supplemented with 1% (v/v) insulin–transferrin–selenium (BD Biosciences), and 1% (v/v) antibiotic/antimycotic solution, 50 mg/ml ascorbate-2-phosphate, 40 mg/ml L-proline, 100 nM dexamethasone, and 1 nM triiodothyronine (all from Sigma). After that, the activity of alkaline phosphatase (AP), a marker of chondrocyte hypertrophy, was assayed using a BCIP/NBT AP color development kit (Beyotime Institute of Biotechnology). DAPI was used to stain nuclei. The AP-positive area and intensity in 5 randomly selected fields viewed under an invert microscope (Leica) were measured using Image Pro Plus 6.0 (Media Cybernetics, Rockville, MD, USA). Relative intensity of AP staining was normalized to cell number.

### Pharmacokinetics study of wogonoside in rat

Pharmacokinetics of wogonoside was conducted in SD rats (200-250g) by intragastric administration at dose of 20 mg/kg. This concentration was equal to the dosage of 40mg/kg in mice in our study according to a body surface area normalized methods [48]. The rats were allowed food and water ad libitum before pharmacokinetics experiment. About 200 μL of blood was taken from tail vein of rats at 5 min, 15min, 30 min, 1, 2, 4, 6, 8 h, 10 h, 12 h, 16 h and 24 h post-dose. After centrifugation of blood samples at 13,000 rpm at 4°C for 5 min, plasm samples were transferred into Eppendorf tube. 100 μL of each serum was processed by adding 3-folds excess of methanol containing 10 ul internal standard (IS) solution followed by vortexing for 1 min. The tube was then centrifuged at 13,000 rpm at 4°C for 10 min. Then the supernatant was filtrated through 0.22 m membrane and an aliquot were injected into UPLC-MS/MS for analysis. Quantification was performed in MRM mode of the transitions *m/z* 461.2→285.2 for wogonoside, *m/z* 461.2→285.2 for IS solution respectively. SCIEX Analyst software (version 1.4.2) was used for data acquisition and analysis. The pharmacokinetic parameters were calculated by the non-compartmental analysis of plasma concentration versus time data using the DAS 3.0 software (Mathematical Pharmacology Professional Committee of China, Shanghai, China).

### Animal model

Sixty Ten-week-old C57BL/6 male wild-type (WT) mice were purchased from Animal Center of Chinese Academy of Sciences Shanghai, China. The protocol for animal care and use conformed to the Guide for the Care and Use of Laboratory Animals of the National Institutes of Health and was approved by the Animal Care and Use Committee of WenzhouMedical University. The osteoarthritis was induced by surgical destabilization of the medial meniscus (DMM) as previously described [44]. In brief, after anaesthesia with intraperitoneally injection of 2% (w/v) pentobarbital (40 mg/kg,) the joint capsule of right knee was opened with an incision just medial to the patellar tendon and the medial meniscotibial ligament was transected with microsurgical scissors. As a control, surgery consisting of an arthrotomy without the transaction of medial meniscotibial ligament, was also performed in the left knee joint of mice and the joint was used as sham group. After surgery, the mice were randomly divided into three groups: sham group, vehicle group and wogonoside treatment group. In the wogonoside treatment group, the mice were given wogonoside 40 mg/kg every day via gastric intubation until sacrificed.

### Histopathologic analysis

The mice were sacrificed by an intraperitoneal over-dosage injection of 10% chloral hydrate and the knee joint were harvested on 8 weeks after surgery. The specimens were fixed in n 4% (v/v) neutral paraformaldehyde for 24 h and decalcified in neutral 10% (v/v) EDTA solution for 1 month. Following dehydrated and embedded in paraffin, the tissues were cut into 5-μm sagittal sections. Slides of each joint were stained with safranin O-fast green(S-O) and H&E. The cellularity and morphology of cartilage and subchondral bone were examined by another group of experienced histology researchers in a blinded manner using a microscope, and evaluated by using a Osteoarthritis Research Society International (OARSI) scoring system for medial femoral condyle and medial tibial plateau as described previously [49]. The severity of synovitis was graded using a scoring system which was previously described [50]: Enlargement of the synovial lining cell layer on a scale of 0–3 (0 = 1–2 cells, 1 = 2–4 cells, 2 = 4–9 cells and 3 = 10 or more cells) and density of cells in the synovial stroma on a scale of 0–3 (0 = normal cellularity, 1 = slightly increased cellularity, 2 = moderately increased cellularity and 3 = greatly increased cellularity).

### X-ray imaging method

After 8 weeks of surgery within or not treatment, the animals were given the X-ray examination. X-ray imaging was performed on all mice to evaluate the joint space and calcification changes of cartilage surface using a digital X-ray machine (Kubtec Model XPERT.8; KUB Technologies Inc.). Proper images were obtained in the following settings: 50Kv and 160μA.

### Immunohistochemical examination

The sections embedded in paraffin were deparaffinized and rehydrated and then the endogenous peroxidase activity was need to be blocked by 3% hydrogen peroxide. After that, the sections were incubated with 0.4% pepsin (Sangon Biotech, Shanghai, P.R. China) in 5 m M HCl at 37 °C for 20 min for antigen retrieval. and nonspecific binding sites was blocked by 5% bovine serum albumin for 30 min at room temperature. The sections were then incubated with the primary antibody (anti-HIF-2α, 1:100; anti-Col X, 1:200, anti-MMP-13, 1:200; anti-VEGF-A, 1:100) overnight at 4 °C. Finally, the sections were incubated with an appropriate HRP-conjugated secondary antibody (Santa Cruz Biotechnology, Dallas, TX, USA) and counterstained with hematoxylin. Images were saved using Image-Pro Plus software, version 6.0 (Media Cybernetics, Rockville, MD, USA), and the integral absorbance values were used as indicators of HIF-2α, Col10A1, MMP-13 and VEGF-A expression levels. At least three sections from each specimen were used to analyze the expression of these proteins.

### Statistical analysis

The experiments were at least performed triplicately. The results were presented as mean ± S.D. Statistical analyses were performed using SPSS statistical software program 20.0. Data were analyzed by one-way analysis of variance (ANOVA) followed by the Tukey’s test for comparison between control and treatment groups. Nonparametric data (Pfirrmann grading) were analyzed by the Kruskal–Wallis H test. P values less than 0.05 were considered significant.

## Abbreviations

OA: osteoarthritis
ECM: extracellular matrix
PGE_2_: prostaglandin E2
NO: nitric oxide
COX-2: cyclooxygenase-2
iNOS: inducible nitric oxide synthase
ADAMTS: thrombospondin motifs
MMPs: metalloproteases
NF-κB: nuclear factor kappa B
IKK: I-κB kinase
PI3K: phosphatidylinositol-3-kinase
HIF-2α: hypoxia-inducible factors two alpha
VEGF: vascular endothelial growth factor
NSAIDs: non-steroidal anti-inflammatory drugs
BSA: bovine serum albumin
DAPI: diamidinophenyl-indole
CMC: carboxymethylcellulose
IL-1β: interleukin-1 beta
TNF-α: tumor necrosis factor alpha
IL-6: interleukin-6
PBS: phosphate-buffered saline
DMM: destabilization of the medial meniscus
